# MITOFIT: A FORMATIVE/SUMMATIVE EVALUATION OF A NOVEL INTERVENTION ON MITOCHONDRIAL FITNESS

**DOI:** 10.1093/geroni/igae098.0261

**Published:** 2024-12-31

**Authors:** Cathy Maxwell, Brandon Grubbs, Jeffrey Boon, John Dunavan, Kelly Knickerbocker, Maulik Patel

**Affiliations:** University of Utah College of Nursing, Salt Lake City, Utah, United States; Middle Tennessee State University, Murfreesboro, Tennessee, United States; Vanderbilt University Medical Center, Nashville, Tennessee, United States; Peer-Tree, Franklin, Tennessee, United States; Vanderbilt University, Nashville, Tennessee, United States; Vanderbilt University, Nashville, Tennessee, United States

## Abstract

A key driver that leads to age-associated decline and chronic disease is mitochondrial dysfunction. Our prior work revealed strong community interest in the concept of mitochondrial fitness that led to development of a video-based intervention to prompt behavior change in adults aged 50+.

**Aim:**

To conduct formative and summative evaluations of MitoFit, an instructional, biologically-based communication intervention aimed at improving physical activity (PA) in older adults, aged 50+.

**Methods:**

Phase 1 formative evaluation- Community-dwelling older adults (N=101), rated the acceptability, appropriateness and helpfulness of our MitoFit video series, titled, “How to Slow Down Aging Through Mitochondrial Fitness.” (4 out of five on a Likert-scale survey). Phase II summative evaluation- A subgroup of phase I participants (N=19) participated in a 1-month MitoFit intervention prototype to evaluate intervention and data collection feasibility (70% completion).

**Results:**

Phase I: Participants (mean age: 67.8 [SD 8.9]; 75% female) rated the MitoFit videos as acceptable (agree: 97%-100%), appropriate (agree: 100%) and helpful (agree: 95%-100%) to support adaptation and continued work on our novel approach. Phase II: Participants (mean age: 71.4; 72% female) demonstrated MitoFit competencies (obtaining pulse, calculating maximum and zone 2 heart rate, demonstration of exercises). At one-month post-instruction, 13 participants (68.4%) had completed a self-initiated daily walking/exercise plan and submitted a daily activity log. Feasibility scores ranged from 89.4% to 94.7%. Fifteen participants (78.9%) stated an intention to continue the MitoFit intervention.

**Conclusion:**

MitoFit was enthusiastically embraced, and is a cost-effective, scalable, and efficacious intervention to advance with community-dwelling adults.

